# Mice deficient in the anti-haemophilic coagulation factor VIII show increased von Willebrand factor plasma levels

**DOI:** 10.1371/journal.pone.0183590

**Published:** 2017-08-24

**Authors:** Klytaimnistra Kiouptsi, Alexandra Grill, Amrit Mann, Mareike Döhrmann, Maren Lillich, Sven Jäckel, Frano Malinarich, Henning Formes, Davit Manukyan, Saravanan Subramaniam, Avinash Khandagale, Cornelia Karwot, Serge C. Thal, Markus Bosmann, Inge Scharrer, Kerstin Jurk, Christoph Reinhardt

**Affiliations:** 1 Center for Thrombosis and Hemostasis (CTH), University Medical Center Mainz, Mainz, Germany; 2 German Center for Cardiovascular Research (DZHK), Partner Site RheinMain, Mainz, Germany; 3 Institute of Clinical Chemistry and Laboratory Medicine, University Medical Center Mainz, Mainz, Germany; 4 Department of Anesthesiology, University Medical Center Mainz, Mainz, Germany; Institut d'Investigacions Biomediques de Barcelona, SPAIN

## Abstract

Von Willebrand factor (VWF) is the carrier protein of the anti-haemophilic Factor VIII (FVIII) in plasma. It has been reported that the infusion of FVIII concentrate in haemophilia A patients results in lowered VWF plasma levels. However, the impact of *F8*-deficiency on VWF plasma levels in *F8*^*-/y*^ mice is unresolved. In order to avoid confounding variables, we back-crossed *F8*-deficient mice onto a pure C57BL/6J background and analysed VWF plasma concentrations relative to C57BL/6J WT (*F8*^*+/y*^) littermate controls. *F8*^*-/y*^ mice showed strongly elevated VWF plasma concentrations and signs of hepatic inflammation, as indicated by increased TNF-α, CD45, and TLR4 transcripts and by elevated macrophage counts in the liver. Furthermore, immunohistochemistry showed that expression of VWF antigen was significantly enhanced in the hepatic endothelium of *F8*^*-/y*^ mice, most likely resulting from increased macrophage recruitment. There were no signs of liver damage, as judged by glutamate-pyruvate-transaminase (GPT) and glutamate-oxalacetate-transaminase (GOT) in the plasma and no signs of systemic inflammation, as white blood cell subsets were unchanged. As expected, impaired haemostasis was reflected by joint bleeding, prolonged *in vitro* clotting time and decreased platelet-dependent thrombin generation. Our results point towards a novel role of FVIII, synthesized by the liver endothelium, in the control of hepatic low-grade inflammation and VWF plasma levels.

## Introduction

Haemophilia A is a rare X-linked recessive bleeding disorder that is caused by the deficiency or absence of FVIII [1]. In about 50% of patients the intron 22 inversion of the factor 8 gene is causative for the disease [2]. Since it has a prevalence of approximately 1 per 6,000 males and the etiology in the normal population is heterogeneous, it is hard to study the pathophysiology of haemophilia A or its co-morbidities with patient samples.

We have used the haemophilia A mouse model with disrupted exon 16 of the *F8* gene for our study [3]. As VWF plasma levels have been demonstrated to be influenced by the genetic background of mice [4], we back-crossed the *F8*^*-/-*^ mouse line onto a pure C57BL/6J genetic background using a speed congenics approach. While in haemophiliacs spontaneous bleeding is frequently observed in the joints (haemarthrosis) and in the musculature (haematomas), *F8*-deficient mice do not show spontaneous bleeding episodes. Nevertheless, well-defined bleeding models, such as the needle-induced knee bleeding model, have been developed to study haemophilia and anti-haemophilic therapies in mice [5].

During coagulation, FVIII *in vivo* is activated through limited proteolysis by thrombin and binds FIXa to accelerate the formation of FXa in the so-called Xase complex, thus amplifying thrombin generation yielding in quantitative formation of fibrin [6]. The anti-haemophilic coagulation FVIII is synthesized in the liver sinusoidal endothelium [7]. By tissue-specific gene targeting approaches it was recently demonstrated not only to be synthesized in, but also stored together with von Willebrand Factor (VWF) in Weibel-Palade bodies of liver endothelial cells [8, 9]. As a marker of endothelial cell activation, plasma VWF is an established endothelial acute-phase response protein with adhesive properties to leukocytes and recognized roles in leukocyte recruitment in different vascular inflammatory diseases [10–12]. While VWF is well-recognized as the carrier molecule of FVIII in plasma, stabilizing FVIII activity by preventing proteolytic degradation [13, 14], the possible impact of FVIII on VWF plasma levels is poorly resolved. Biochemical studies have reported a synergistic role of FVIII and platelets on the cleavage of VWF multimers by ADAMTS13 [15–17]. In spite of these mechanistic insights from *in vitro* analyses and the wealth of studies on haemophilia A, the significance of genetic *F8*-deficiency, as it occurs in haemophilia A, on VWF plasma levels and immune cell infiltration of the liver is unexplored.

To systematically investigate the role of FVIII on VWF content in the liver endothelium, on VWF plasma levels, and VWF multimer size *ex vivo*, and to determine its implication in hepatic inflammation, we have used the *F8*-deficient haemophilia A mouse model [3] on a pure C57BL/6J background. In addition to the haemostatic and bleeding characteristics, we analyzed littermates from heterozygous breedings for their hepatic endothelial VWF content, VWF plasma levels and multimer size. Interestingly, analysis of *F8*-deficient mice revealed elevated VWF content in the liver endothelium along with signs of low-grade hepatic inflammation and increased VWF plasma levels compared with their co-housed WT littermate controls.

## Materials and methods

### Animals

C57BL/6J WT and B6;129S4-F8^tm1Kaz^/J were purchased from Jackson Laboratory (Bar Harbor, Me, USA). The B6;129S4-F8^tm1Kaz^/J were backcrossed according to marker assisted selection breeding (speed congenic) to the C57BL/6J background by Taconic biosciences (Albany, New York, USA). Only male C57BL/6J WT and C57BL/6J F8^-/y^ or C57BL/6J F8^+/y^ mice from heterozygous breedings were used for this study. All experimental animals were 8–14 weeks old and housed in conventional cages (max 5 mice per cage) with a 12h light-dark cycle. All experiments performed in mice were approved by the local committee on legislation on protection of animals (Landesuntersuchungsamt Rheinland-Pfalz, Koblenz, Germany; 23 177–07 / A12-1-006, 23 177–07 / G11-1-045, 23 177-07/G16-1-013)

### Blood collection for blood cell counts and liver enzymes

Whole blood was collected by cardiac puncture. For differential blood analysis blood was transferred to EDTA tubes, for GOT and GPT measurements heparinized tubes were used. Both analyses were performed by the Insitute of Clinical Chemistry and Laboratory Medicine at University Medical Center Mainz.

### Joint bleeding model

Mice were injected subcutaneously with Buprenorphine (0.1mg/kg BW) (Temgesic Vet, Berkshire, UK) 30 min prior to the experiment. They were anesthetized intraperitoneally with midazolam (5 mg/kg BW) (Hameln, Germany) and medetomidine (0.5 mg/kg BW) (Dorbene Vet, Zoetis, Berlin, Germany). The fur was removed from both legs. The external joint diameter was calculated as the mean of three independent measurements per leg by a Mitutoyo thickness measuring system (Takatsu-ku, Kawasaki, Kanagawa, Japan). Joint bleeding was induced to the right knee by transcutaneous insertion of a 30 G needle through the patellar ligament and into the infrapatellar fat pad, avoiding contact with the cartilage and bone. Subsequently, the needle was withdrawn gently. Left knee served as an intra-animal control. 0.1 mg/kg BW Buprenorphine was administrated subcutaneously at 6 h after injury and was continued 3- times per day, for the following 2 days. On the second experimental day, mice were anesthetized as described above and the weight and the external joint diameter were determined. Directly thereafter the mice were euthanized.

### Thromboelastography

Citrated (3.8%) whole blood was collected by cardiac puncture. Blood was recalcified with 20 mM Ca^2+^ directly prior to measurement (50 μl of Ca^2+^-Hepes solution; 100 mM CaCl_2_ + 1 mM Hepes). Reconstitution of the FVIII activity was succeeded by adding 2.5 U/ml human recombinant FVIII. Clotting time and clot formation time were measured by a whole blood haemostasis analyzer (ROTEM delta, Tem GmbH, Munich, Germany).

### Calibrated automated thrombography

Citrated blood was collected by cardiac puncture, diluted with one volume Tyrode’s buffer (5.5 mM D-glucose, 140 mM NaCl, 12 mM NaHCO_3_, 2.7 mM KCl, 0.42mM Na H_2_PO_4_, 5 mM Hepes, pH 7.4) and centrifuged at 100 x g for 4 min at room temperature to obtain platelet-rich plasma (PRP). Platelet-poor plasma (PPP) was prepared by centrifugation of residual PRP including the buffy coat at 30’000 x g for 10 min at room temperature. Thrombin generation was determined in PRP triggered by tissue factor (1 pM tissue factor, PRP-reagent) or by α-thrombin (Sigma, St. Louis, USA) (0.1 U/mL) and in PPP triggered by tissue factor in the presence of phospholipids (5 pM tissue factor, PPP-reagent) with fluorogenic calibrated automated thrombography, FluCa-Kit (Thrombinoscope BV, Maastricht, The Netherlands).

### Quantitative real-time PCR

Total RNA was isolated from liver and lung tissues with the RNeasy Kit (Qiagen, Hilden, Germany) according to manufacturer’s instructions and was then reverse transcribed (High Capacity cDNA Reverse Transcription Kit; Applied Biosystems, Foster City, USA). Total RNA (2 μg) was reverse transcribed with the High Capacity cDNA Reverse Transcription Kit (Applied Biosystems, Foster City, USA) and SYBR green-based qRT–PCR was performed with iQ SYBR Green Supermix (Bio-Rad Laboratories, Hercules, CA, USA). The oligonucleotide sequences used are listed in Table 1.

**Table 1:** 

F8_for	coagulation factor 8	ATGCAAATAGCACTCTTCGCT
F8_rev	coagulation factor 8	ACACTGAGCAGATCACTCTGA
VWF_for	von Willebrand Factor	CTTCTGTACGCCTCAGCTATG
VWF_rev	von Willebrand Factor	GCCGTTGTAATTCCCACACAAG
st3gal4_for	ST3 Beta-Galactoside Alpha-2,3-Sialyltransferase 4	ACCAGCAAATCTCACTGGAAG
st3gal4_rev	ST3 Beta-Galactoside Alpha-2,3-Sialyltransferase 4	CCCTGGAAGCATGGCTCTTTC
CD45_for	Leucocyte marker	ATGGTCCTCTGAATAAAGCCCA
CD45_rev	Leucocyte marker	TCAGCACTATTGGTAGGCTCC
TNFalpha_for	Tumor necrosis factor α	CCAGACCCTCACACTCA
TNFalpha_rev	Tumor necrosis factor α	CACTTGGTGGTTTGCTACGAC
TLR4_for	Toll like receptor 4	ATGGCATGGCTTACACCACC
TLR4_rev	Toll like receptor 4	GAGGCCATTTTTGTCTCCACA
SAA3_for	Serum amyloid A 3	TGCCATCATTCTTTGCATCTTGA
SAA3_rev	Serum amyloid A 3	CCGTGAACTTCTGAACAGCCT
F4/80_for		CTCTGTGGTCCCACCTTCAT
F4/80_rev		GATGGCCAAGGATCTGAAAA
CLEC4M_for	C-type lectin domain family 4 member M	AGACACAGCAAGTGGTCATTC
CLEC4M_rev	C-type lectin domain family 4 member M	GTTGCGGCTCTGCTTCGTA
ASGR1_for	Asialoglycoprotein receptor 1	CTGGGTGGAGTATGAAGGCAG
ASGR1_rev	Asialoglycoprotein receptor 1	GTCAGTTAGGCCAATCCAAGTG
ASGR2_for	Asialoglycoprotein receptor 2	CTGCAAGAAGAGTTTCGGACC
ASGR2_rev	Asialoglycoprotein receptor 2	GTATGGCGTTTGTGCTACCTC
SiglecF_for	Sialic acid-binding immunoglobulin-like lectin F	GAAGGAAGCTCAAGGTCGATTC
SiglecF_rev	Sialic acid-binding immunoglobulin-like lectin F	TGTGTCGATTTTCTGTGCATCT
LRP1_for	Low density lipoprotein receptor-related protein 1	GACCAGGTGTTGGACACAGATG
LRP1_rev	Low density lipoprotein receptor-related protein 1	AGTCGTTGTCTCCGTCACACTTC

### Immunohistochemistry

Tissues were fixed in Roti^®^-Histofix 4% (Carl Roth GmbH + Co. KG, Karlsruhe, Germany) and provided to the Core Facility Histology of the University Medical Center Mainz for F4/80 and VWF staining (Dako, Glostrup, Denmark). Paraffin sections were routinely stained with haematoxylin eosin. All sections were analyzed by light microscopy (Axia Lab.A1, Zeiss, Oberkochen, Germany) and scored in a blinded fashion by three different experts.

### FVIII injections

50 U/kg BW of recombinant FVIII (Kogenate, Bayer, Leverkusen, Germany) or equal volume of 0.9% NaCl were injected into the lateral tail vein of *F8*^*-/y*^ mice. The *F8*^*+/y*^ remained untreated. Whole blood was collected from the orbital sinus in EDTA tubes 2h after the injections and centrifuged at 800 x g for 10 min to aquire PRP. For platelet poor plasma (PPP), PRP was centrifuged at 1.200 x g for 10 min.

### Von Willebrand factor ELISA

A 96 well plate was coated overnight with anti-human VWF antibody (Dako, Glostrup, Denmark, 1:1000 dilution) and was then blocked for 1 hour with 1% BSA in PBS pH 7.4. Blood was collected from the facialis vein in a K2 EDTA microvette tube (Sarstedt Nümbrecht, Germany) and PPP was prepared by centrifugation at 800 x g for 10 min followed by centrifugation at 1.200 x g for 10 min. A standard curve was created from increasing dilutions of pooled human EDTA plasma with defined VWF concentration obtained from 10 healthy blood donors. PPP and standards were incubated for 1 h followed by 3 washing steps with 0.2% Tween in PBS. The polyclonal rabbit anti-human VWF/HRP was incubated for 1 h (Dako, Glostrup, Germany, 1:1000) and the plate was washed 3 times. 80 μl of the chromogenic substrate TMB (Sigma) were added and the reaction was terminated by adding 80 μl 2N H_2_SO_4_. The absorbance was measured at 450nm by an Opsys MR microplate reader (Dynex technologies,USA).

### Von Willebrand factor multimer analysis

Frozen citrated plasma samples were thawed in a 37°C water bath for 10 min and gently mixed. Thawed plasma samples (30 μL) were diluted in 30 μl of 2x sample buffer (0.02 mol/l Tris, 0.002 mol/l EDTA, pH 8.0, with 2% SDS), 130 μl of 1x sample buffer (0.01 mol/l Tris, 0.001 mol/l EDTA, pH 8.0, with 2% SDS) and supplemented with 10 μl of 0.2% solution of sodium bromphenol blue. They were incubated 30 min at 58°C in a water bath and centrifuged for 1 min at full speed. Electrophoresis samples were maintained at RT until loading. SeaKam HGT (P) high gelling temperature agarose (Lonza Verviers, Verviers, Belgium) was used to prepare a stacking gel (0.75% agarose, 0.1% SDS, 0.125 mol/l Tris, pH 6.8) and 2-Hydroxyethyl agarose (Sigma-Aldrich, St. Louis, MO) was used for the separating gel (1% agarose, 0.1% SDS, 0.75 mol/l Tris, pH 8.8). A horizontal slab gel electrophoresis unit (LKB 2117/Pharmacia Biotech Multiphor Electrophoresis Systems, Uppsala, Sweden) was water-cooled to 6°C prior to and throughout electrophoresis. Agarose gels were placed on the horizontal ceramic electrophoresis cooling slab. Filter paper wicks, immersed in tanks containing electrophoresis running buffer (0.05 mol/l Tris, 0.384 mol/l glycine with 0.1% SDS) were placed on the cathode (sample) and anode ends of each agarose slab. Plasma samples (45 μl) were loaded on the agarose gel sample wells. Electrophoresis began at constant current (13 mA) for approximately 2 h to move the plasma proteins into the stacking gel. The wells were then sealed with additional stacking gel and electrophoresis resumed at constant current of 15 mA and 1–3 W overnight, until termination of electrophoresis when the dye front reached the anodal wick. After stopping electrophoresis (day 2), gels were incubated for 15 min in electrophoresis running buffer before transfer to nitrocellulose membrane by wet tank blotting for 3 h at constant current (1 A) in blotting buffer (0.008 mol/L Na2HPO4). The membrane was blocked for 1 h with blocking buffer (3% bovine serum albumin, 0.1% Tween in TBS) at RT and incubated in rabbit anti-human VWF (Dako A0082, 1:1000 in blocking buffer) over night at 4°C. After 3x washing (0.1% Tween in TBS) membrane was incubated with AP-conjugated goat anti-rabbit antibody (Dako D0487, 1:1000 in washing buffer) for 2 h at RT. After 3x washing, separated multimers were visualized using a buffered solution containing BCIP/NBT, pH 9.5 (SIGMA FAST^™^ BCIP/NBT, Sigma-Aldrich).

### ADAMTS-13 ELISA

The ADAMTS-13 activity was measured by the Actifluor ADAMTS-13 activity assay according to manufacturer’s instructions (Sekisui Diagnostics, Lexington, USA). Briefly, citrated blood was collected by cardiac puncture and platelet poor plasma was prepared as described previously. Assay buffer (provided) was added in each well of a 96 well plate followed by samples and standards (provided). The plate was warmed for 3 min before addition of the ALEXA488-vWF86 FRET substrate. The plate was read with excitation at 485 nm, emission at 535nm and cut off filter at 530 nm by a fluoroskan ascent FL (Thermo Scientific, USA). Afterwards, it was incubated for 20 min at 37°C and read once more with the same settings. The net charge in fluorescence was calculated (ΔRFU) as RFU_T = 20_-RFU_T = 0_). A standard curve was constructed by plotting the ΔRFU for each standar4d versus the corresponding concentration.

**Sialic acid ELISA** was purchased from MyBioSource (San Diego, CA, USA) and performed according to manufacturer’s instructions with undiluted EDTA plasma samples.

### Treatment of *F8*^*-/y*^ mice with sialylation inhibitor 2,4,7,8,9-pentaacetyl-3Fax-Neu5Ac-CO2Me (3F-NeuAc)

Sialylation inhibitor 2,4,7,8,9-pentaacetyl-3Fax-Neu5Ac-CO2Me (3F-NeuAc) was purchased from Merck Millipore (Darmstadt, Germany). The inhibitor was dissolved in DMSO and diluted 1:5 in NaCl to a volume of 100 μl. *F8*^*-/y*^ mice were injected twice on alternate days for 4 days with 3F-NeuAc (Inhibitor) at a dosage of 100 mg/kg BW in the lateral tail vein. WT animals in the control group received 20 μl DMSO solved in NaCl to a volume of 100 μl following the same injection protocol. Afterwards whole blood was collected from the orbital sinus, transferred to EDTA tubes and platelet poor plasma was prepared as described previously. PPP was analyzed by VWF and sialic acid ELISA kit.

### Statistical analysis

All data and statistical analysis were performed using GraphPad Prism 6 for Windows (GraphPad Software, San Diego, CA, USA). Data are presented as mean values ± standard error of the mean. Statistical analyses were performed using the student’s unpaired *t*-test, one-way analysis of variance (ANOVA), or two-way ANOVA. * p< 0,05, ** p<0,01, ***p<0,001.

## Results

### F8-deficient mice mimic the haemophilia A phenotype

**Fig 1:**
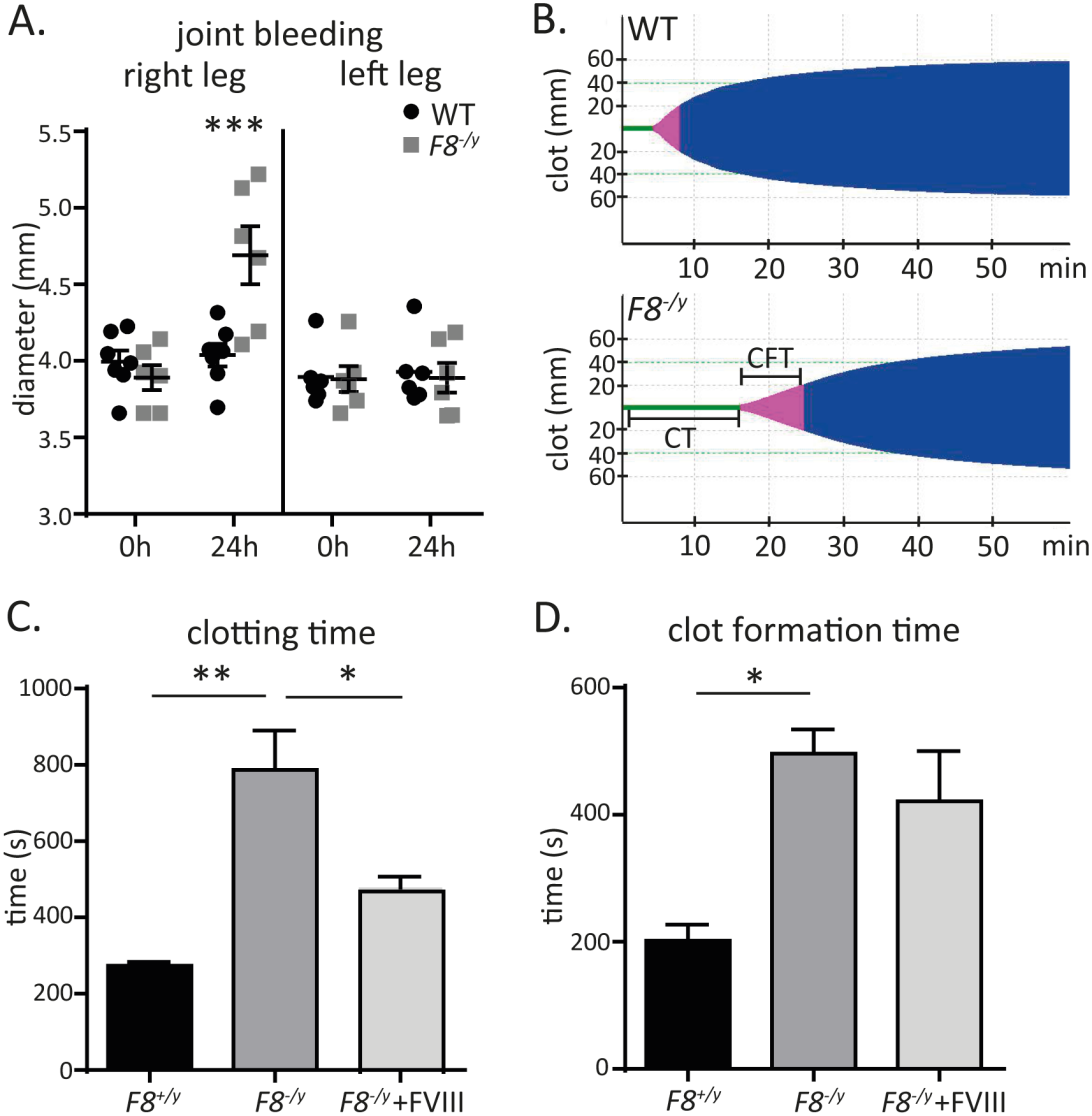
Bleeding phenotype of the *F8*^*-/y*^ mice. (**A**) Patella diameter before and 24h after joint bleeding induction to the right knee (n = 7,6) of C57BL/6J and *F8*^*-/y*^ with the left knee (n = 6,6) set as a control. (**B**) Representative rotational thromboelastometry (ROTEM) graphs of a C57BL/6J and a *F8*^*-/y*^, illustrating clotting time (green), clot formation time (pink) and clot firmness (blue). (**C**) Clotting times and (**D**) clot formation times of C57BL/6J, *F8*^*-/y*^, and *F8*^*-/y*^ blood samples reconstituted with 2.5 U/ml of human recombinant FVIII (n = 3). All data were expressed as means ± SEM. Statistical comparisons were performed using one-way ANOVA or two-way ANOVA * p< 0.05, ** p<0.01, ***p<0.001.

The *F8*-deficient mouse [3] is a genetic model for haemophilia A. The counts of white blood cells, specifically neutrophils, monocytes, eosinophils, basophils, and lymphocytes, as well as the numbers of red blood cells and hemoglobin concentration were not different between *F8*^*+/y*^ (WT) and *F8*^*-/y*^ (*F8*-deficient) mice (S1A–S1I Fig), excluding acute inflammatory processes in those mice. In the needle-induced knee joint bleeding model [18], *F8*-deficient mice showed a 20% increase in joint swelling diameter compared with littermate WT controls at 24 hours after puncture (Fig 1A). In thromboelastometry, citrated whole blood from mice deficient in the anti-haemophilic FVIII (*F8*^*-/y*^) was characterized by significantly prolonged clotting time (CT) as well as increased clot formation time (CFT) (Fig 1B). The measured CT of *F8*^*-/y*^ whole blood was three times longer than in *F8*^*+/y*^ control mice and was partially rescued by addition of human recombinant FVIII (Kogenate) at a concentration of 2.5 U/ml (Fig 1C). Also the CFT of *F8*^*-/y*^ whole blood was 2 to 3 times prolonged compared with *F8*^*+/y*^ controls (Fig 1D). Our results clearly demonstrate that the *F8*-deficient mice on a C57BL/6J genetic background display the expected bleeding phenotype.

### Platelets from F8 deficient mice exhibit impaired thrombin generation

**Fig 2:**
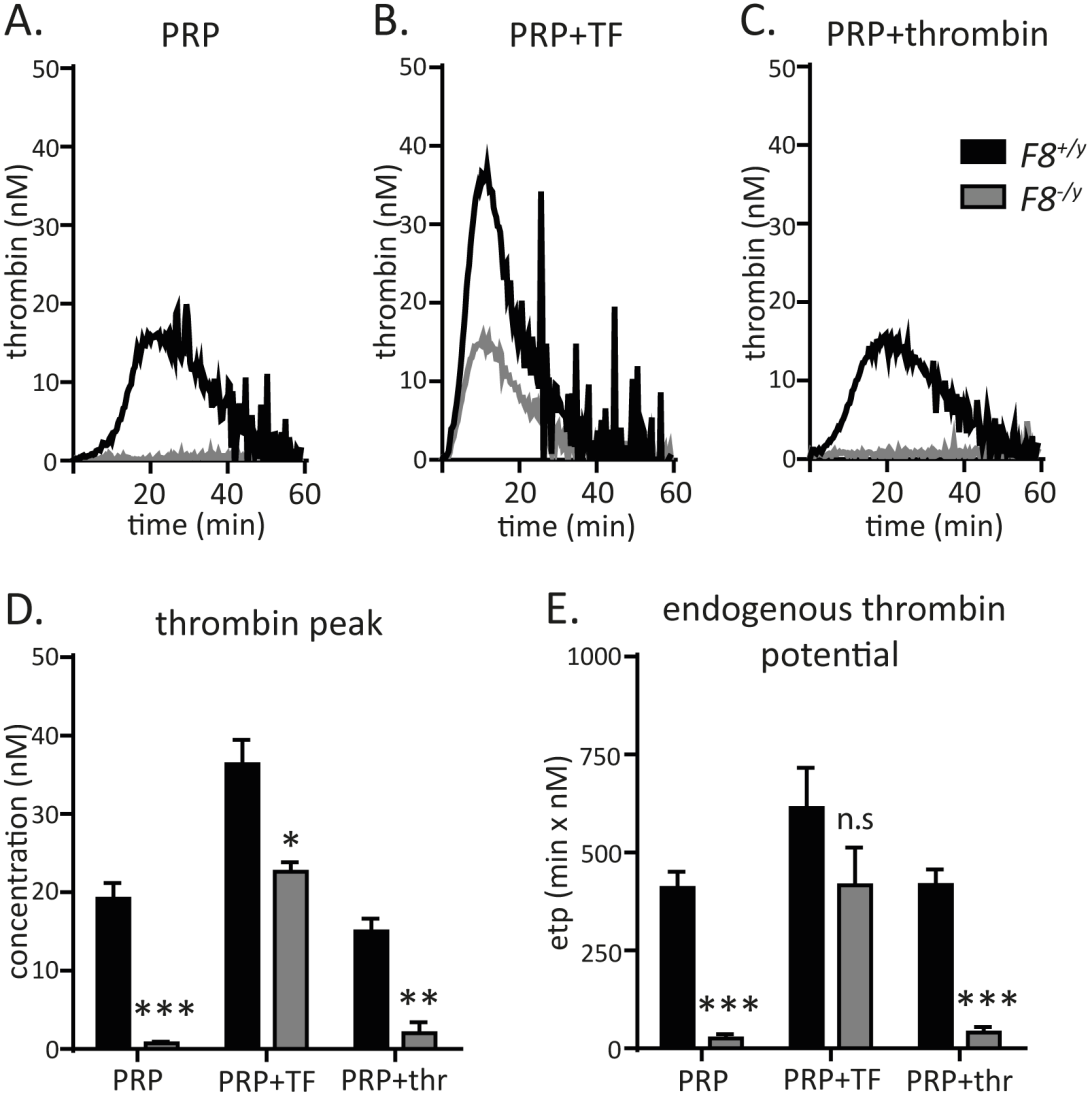
Impaired thrombin generation by platelets from *F8*^*-/y*^ mice. (**A**), (**B**) and (**C**) Representative thrombograms of a *F8*^*+/y*^ (WT) vs a *F8*^*-/y*^ mouse. Thrombin generation in unstimulated platelet-rich plasma (PRP) (**A**), in PRP induced by 1 pM tissue factor (TF) (**B**), and in PRP induced by 0.1 U/ml thrombin (thr) (**C**), respectively. (**D**) Thrombin peak in unstimulated PRP (n = 5,2), PRP plus 1 pM TF (n = 5,2), and 0.1 U/ml thr-stimulated PRP (n = 5,2). (**E**) Endogenous thrombin potential in unstimulated PRP (n = 5,2), PRP triggered by 1 pM TF (n = 5,2), and 0.1U/ml thr-stimulated platelets in PRP (n = 5,3), analyzed by calibrated automated thrombography of *F8*^*+/y*^ vs *F8*^*-/y*^ mice. All data were expressed as means ± SEM. Statistical comparisons were performed using the Student’s *t*-test, * p< 0.05, ** p<0.01, ***p<0.001.

F8-deficiency resulted in nearly complete loss of thrombin generation not only in platelet-rich plasma (PRP), but also when either 1 pM tissue factor (TF) or 0.1 U/ml thrombin (thr) was used as a trigger. The thrombin peak and the endogenous thrombin potential (etp) were strongly reduced in *F8*^*-/y*^ mice compared to *F8*^*+/y*^ controls (Fig 2A–2E). Collectively, these analyses demonstrate the critical role of FVIII in platelet-dependent amplification of thrombin generation triggered by a low to moderate concentration of TF and thrombin.

### *F8*-deficient mice show low-grade inflammation in the liver

**Fig 3:**
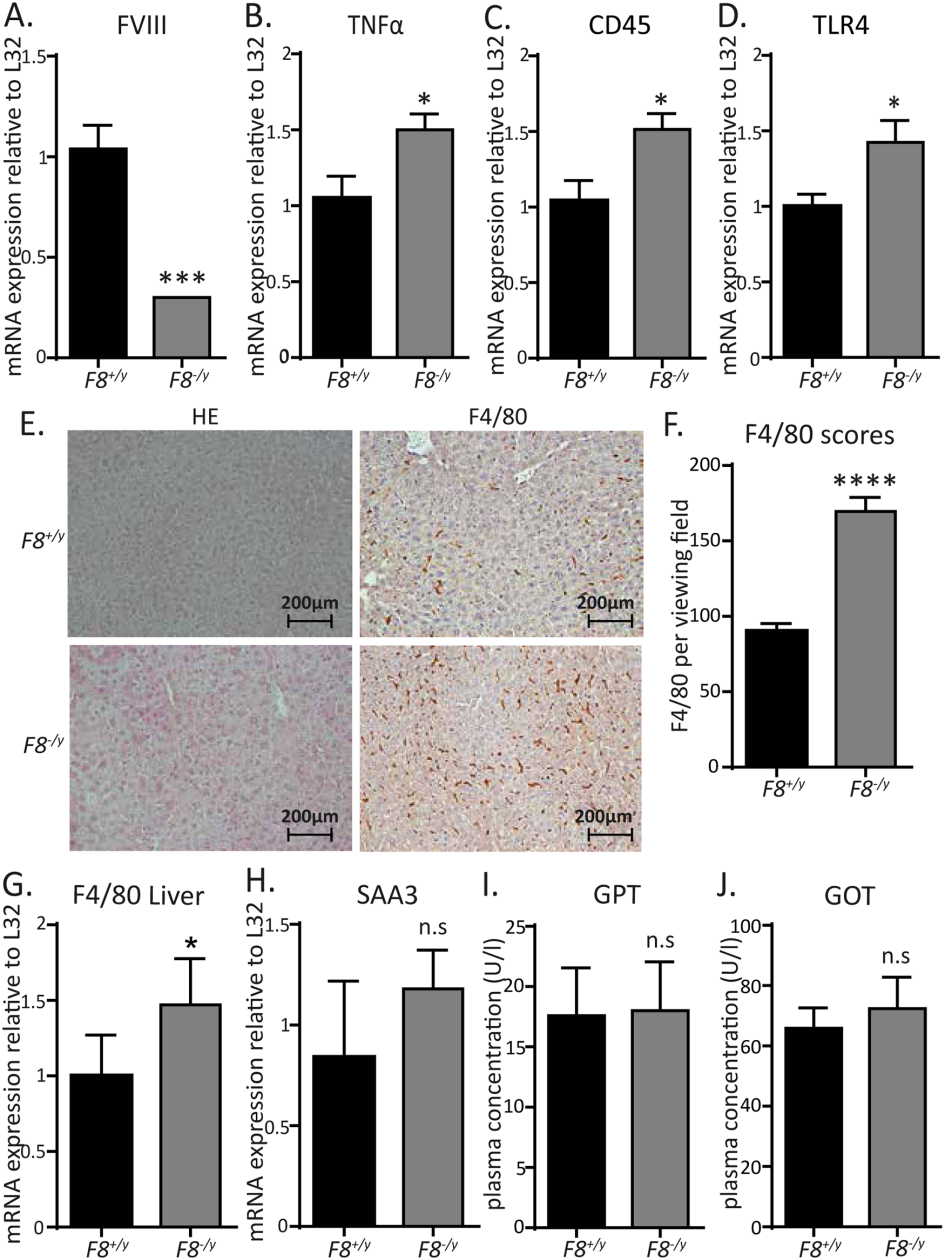
Low-grade inflammatory phenotype in the liver of *F8*^*-/y*^ mice. (**A**) FVIII (n = 5,4), (**B**) TNFα (n = 6,7), (**C**) CD45 (n = 7), and (**D**) TLR4 (n = 6,4) hepatic transcript levels of *F8*^*+/y*^ (WT) vs *F8*^*-/y*^ mice. (**E**) Representative hepatic tissue sections of *F8*^*+/y*^ vs *F8*^*-/y*^ stained with anti-mouse F4/80 antibody (200x magnification). (**F**) Visual scoring of the F4/80 stained hepatic tissue sections (n = 5). (**G**) Hepatic transcript levels of the macrophage marker F4/80 in *F8*^*+/y*^ (WT) vs *F8*^*-/y*^ mice (n = 4,7). (**H**) Hepatic SAA3 transcript levels in *F8*^*+/y*^ (WT) vs *F8*^*-/y*^ mice (n = 5,5). (**I**) Serum glutamate-pyruvate-transaminase levels in *F8*^*+/y*^ (WT) vs *F8*^*-/y*^ mice (n = 5,5). (**J**) Serum glutamat-oxalacetat-transaminase levels in *F8*^*+/y*^ (WT) vs *F8*^*-/y*^ mice (n = 6,5). All data were expressed as means ± SEM. Statistical comparisons were performed using the Student’s *t*-test, * p< 0.05, ***p<0.001, ****p<0.0001.

To explore potential effects of *F8*-deficiency in highly vascularized organs, we analyzed liver and lung tissues. As expected, FVIII mRNA expression was strongly decreased in the liver of *F8*^*-/y*^ mice relative to *F8*^*+/y*^ controls (Fig 3A). Also the transcripts of the pro-inflammatory cytokine tumor necrosis factor-α (TNF-α), the pan-leukocyte marker CD45, and Toll-like receptor-4 (TLR4) were significantly increased in *F8*^*-/y*^ livers compared to *F8*^*+/y*^ controls (Fig 3B–3D), whereas these were not changed in the lung (data not shown). This is in line with the observed phenotype in hematoxylin/eosin stained tissue sections showing increased counts of F4/80-positive macrophages as determined by immunohistochemistry (Fig 3E and 3F). Furthermore, F4/80 transcripts were elevated in the livers of *F8*^*-/y*^ mice compared with *F8*^*+/y*^ controls (Fig 3G) and the expression of the acute-phase protein serum amyloid A3 (SAA3) showed a slight tendency to increased expression (Fig 3H). In the absence of liver damage, low-grade hepatic inflammation did not lead to elevated liver enzymes in the serum of *F8*^*-/y*^ mice (Fig 3I and 3J). Collectively, our results demonstrate hepatic low-grade inflammation in the livers of *F8*^*-/y*^ mice.

### Endothelial VWF antigen levels are increased in the liver of *F8*-deficient mice

**Fig 4:**
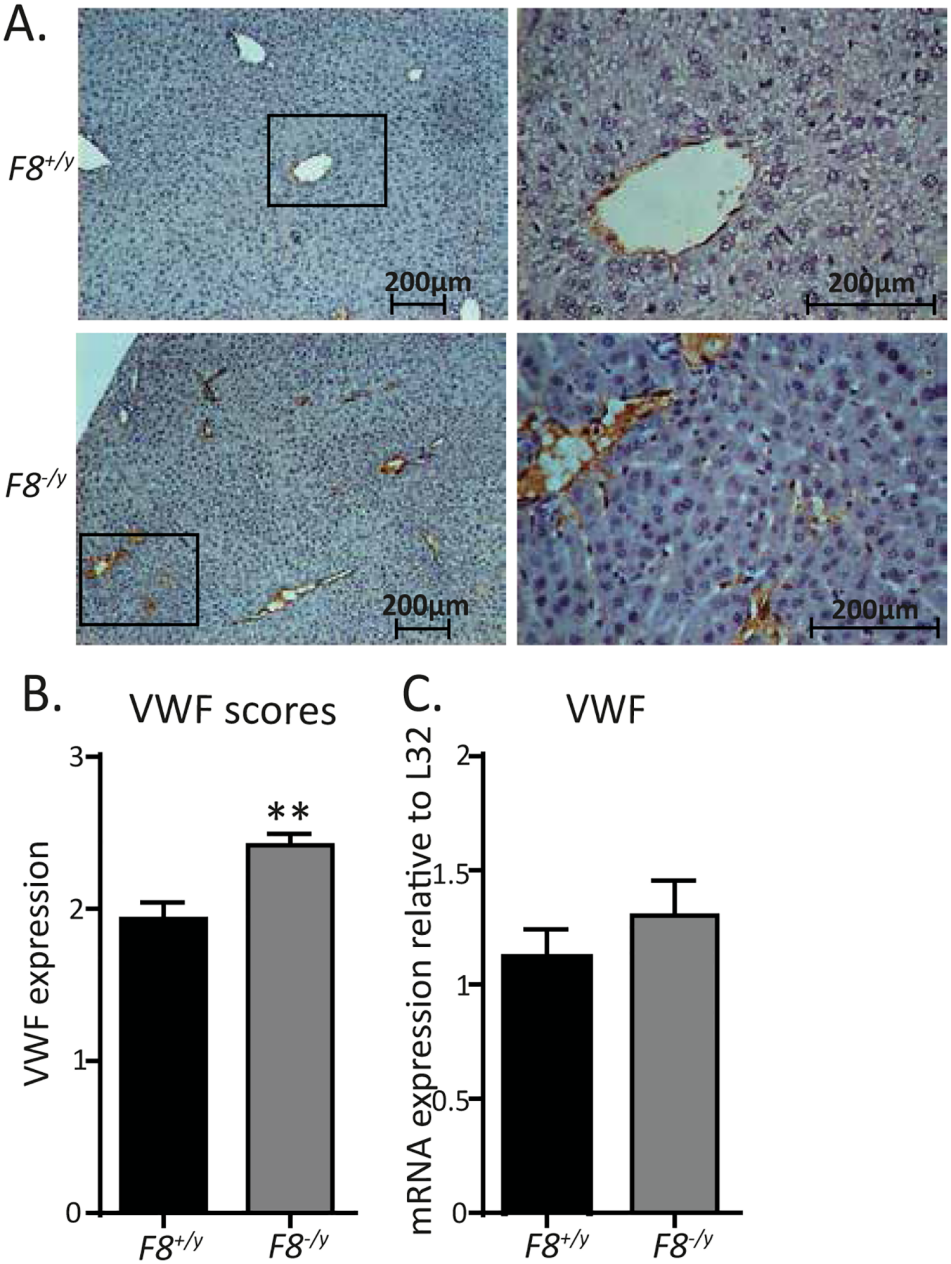
Increased VWF staining of the hepatic endothelium of *F8*^*-/y*^ mice. (**A**) Representative hepatic tissue sections of *F8*^*+/y*^ (WT) vs *F8*^*-/y*^ mice stained with anti-human VWF antibody (100x and 400x magnification). (**B**) Visual scoring of the VWF stained hepatic tissue sections of *F8*^*+/y*^ (WT) vs *F8*^*-/y*^ mice (n = 7,6). (**C**) Hepatic VWF transcript levels in *F8*^*+/y*^ (WT) vs *F8*^*-/y*^ mice (n = 4,7). All data were expressed as means ± SEM. Statistical comparisons were performed using the Student’s *t*-test, ** p<0.01.

As VWF, the carrier protein for FVIII, is an endothelial-derived acute-phase protein that is elevated under inflammatory conditions, we next analyzed hepatic VWF antigen expression. Immunohistochemistry analyses of liver sections from *F8*^*-/y*^ mice revealed enhanced VWF staining as compared to the liver specimens of *F8*^*+/y*^ control mice (Fig 4A and 4B). However, VWF mRNA levels were unchanged between *F8*^*+/y*^ and *F8*^*-/y*^ mice (Fig 4C), suggesting that VWF processing was modulated post-transcriptionally. Hence, our results imply that *F8*-deficiency triggers low-grade inflammation in the liver, which is associated with increased hepatic endothelial VWF antigen.

### VWF plasma levels are elevated in *F8*-deficient C57BL/6J mice

**Fig 5:**
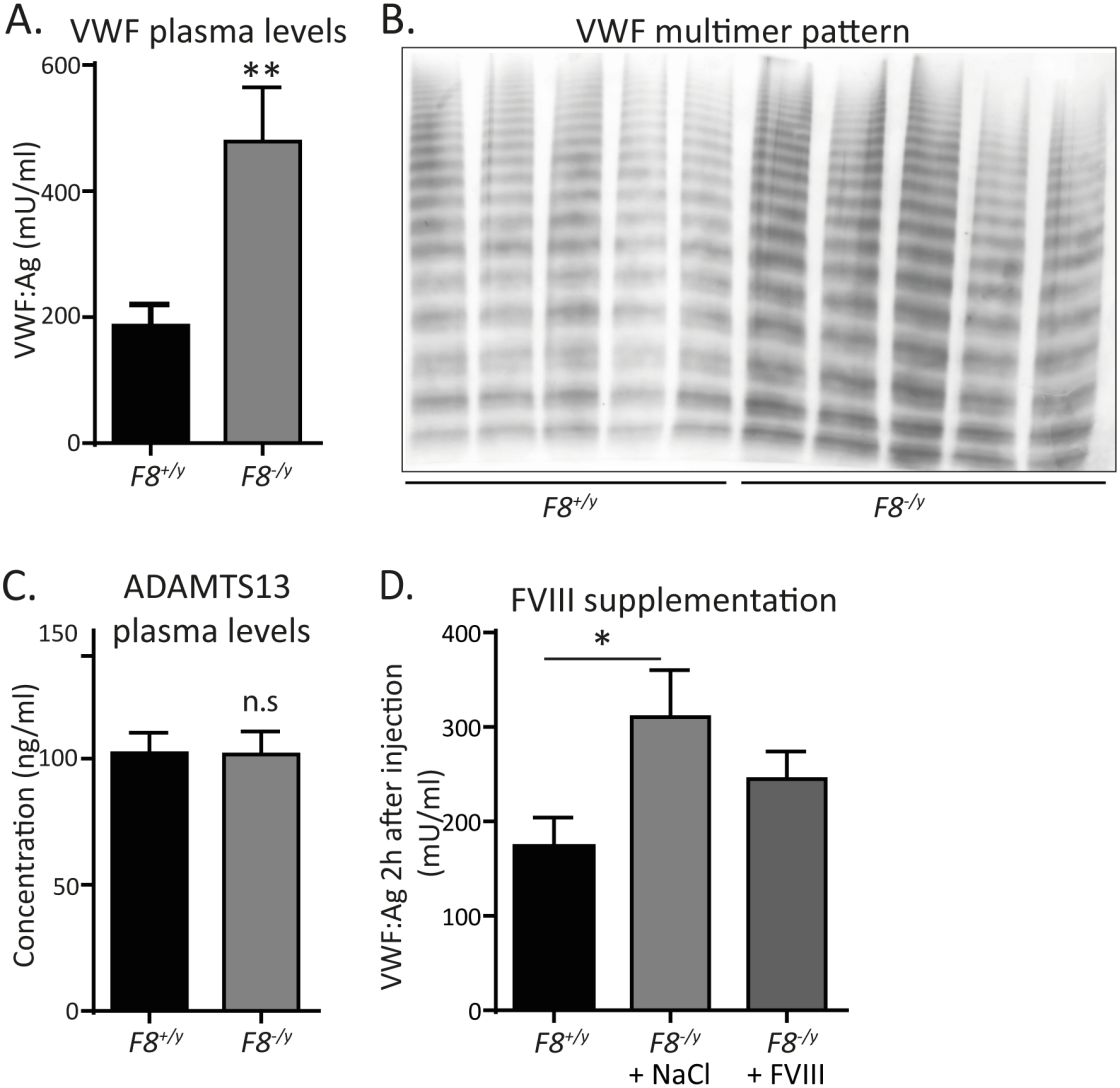
Increased VWF levels in the plasma of *F8*^*-/y*^ mice. (**A**) VWF antigen levels in plasma of *F8*^*+/y*^ (WT) vs *F8*^*-/y*^ mice (n = 9). (**B**) VWF multimer analysis of plasma samples from *F8*^*+/y*^ vs *F8*^*-/y*^ mice (n = 5). (**C**) ADAMTS13 levels in plasma of *F8*^*+/y*^ vs *F8*^*-/y*^ mice (n = 7). (**D**) VWF antigen levels in plasma of *F8*^*-/y*^ mice, 2h after tail vein injection of recombinant FVIII (Kogenate) at a dose of 1.5 U per 30g body weight or with 0.9% NaCl solution as a vehicle control (n = 11,10,12). All data were expressed as means ± SEM. Statistical comparisons were performed using the Student’s *t*-test or one-way ANOVA, * p< 0.05, ** p<0.01.

As immunohistochemistry analyses revealed increased VWF antigen levels in the liver endothelium of *F8*^*-/y*^ mice and because VWF is the carrier molecule that stabilizes FVIII in plasma to increase its half-life [19], we compared VWF plasma levels between *F8*^*-/y*^ mice and co-housed WT littermate controls. In line with a previous report, describing the reduction of VWF plasma levels by FVIII infusion in haemophilia A patients [20], we repeatedly found a 2.5-fold increase in VWF plasma levels in *F8*^*-/y*^ mice relative to *F8*^*+/y*^ littermate controls (Fig 5A). This increase was also recognized in the intensity of the plasmatic VWF multimers (Fig 5B). In line with unchanged ADAMTS13 concentrations in the plasma of *F8*^*-/y*^ mice (Fig 5C), there was no qualitative shift in the VWF multimer pattern (Fig 5B).

While the expression of several known factors, supporting VWF clearance were unchanged in the liver, the mRNA expression of ST3Gal4, a modifier gene counteracting asialoglycoprotein receptor-mediated clearance of VWF [21], was significantly increased in *F8*^*-/y*^ mice (S2A–S2E Fig). In contrast to the liver, no changes in VWF transcripts or ST3Gal4 mRNA expression were found in the lung (S2F–S2H Fig). Treatment with the global sialyltransferase inhibitor 2,4,7,8,9-pentaacetyl-3Fax-Neu5Ac-CO2Me (3F-NeuAc) [22] resulted in a reduction of VWF plasma levels of *F8*^*-/y*^ mice to the levels of WT control mice (S3A Fig), showing that sialylation of VWF is an important determinant of its clearance. However, ELISA measurements indicated that the sialylation of plasma proteins was unchanged in the blood of *F8*^*-/y*^ mice relative to *F8*^*+/y*^ controls (S3B Fig), arguing against a major role of reduced hepatic ST3Gal4 expression and its functional role in sialylation, which might possibly influence VWF plasma levels in *F8*^*-/y*^ mice.

To further pinpoint the involvement of plasmatic FVIII in the regulation of VWF plasma levels, *F8*^*-/y*^ mice were injected via the tail vein either with human recombinant FVIII (Kogenate) at a dose of 1.5 U per 30 g body weight, or with the same volume of 0.9% NaCl solution as a vehicle control. While *F8*^*-/y*^ mice again showed increased VWF plasma levels relative to the untreated *F8*^*+/y*^ controls, we found that substitution of FVIII diminished VWF plasma levels (Fig 5D). Collectively, our results indicate that increased VWF antigen in the liver endothelium of *F8*^*-/y*^ mice is associated with increased VWF plasma levels, which is likely a consequence of the hepatic low-grade inflammation in *F8*^*-/y*^ mice.

## Discussion

The most common complication of haemophilia A is recurrent haemarthrosis in the knee joints that leads to chronic degenerative arthropathy [23]. In the haemophilia A mouse joint bleeding model [24], it has been demonstrated that replacement therapy with FVIII could prevent this bone and soft tissue degeneration [25]. *F8*^-/y^ mice on a C57BL/6J background showed the expected bleeding phenotype with increased knee joint swelling in the needle-induced knee bleeding model compared with WT littermate controls. In addition to increased trabecular thickness 24 hours after needle injury, and in line with previous work [26], we recorded increased clotting times and increased clot formation times in the whole blood of *F8*-deficient mice that could be partially rescued by supplementation of recombinant human FVIII (Kogenate) in murine whole blood. This is due to the crucial role of FVIII in platelet-dependent thrombin generation, when low to moderate concentrations of tissue factor or thrombin are used as a trigger.

With washed platelets, it has been demonstrated that the activation state of platelets from *F8*-deficient mice does not significantly differ from WT littermate control platelets [27]. However, thrombin generation in platelet-poor plasma, induced by low to moderate concentrations of TF, critically depends on the formation of the Xase complex that is hampered in hemophilia A patients [28]. This is reflected by the observed nearly complete loss of platelet-dependent thrombin generation in platelet-rich plasma of *F8*^*-/y*^ mice, induced by low to moderate concentrations of TF or thrombin.

Most interestingly, the back-crossed littermate *F8*-deficient genetic mouse model revealed increased VWF content in the liver endothelium together with increased VWF plasma levels, which was associated with hepatic low-grade inflammation. We found elevated TNF-α, CD45, and TLR4 mRNA levels in the livers of *F8*^*-/y*^ mice that are hallmarks of inflammation in the liver. Moreover, increased F4/80 macrophage counts and increased F4/80 transcript levels were detected in the livers of *F8*^*-/y*^ mice, indicating that *F8*-deficiency results in a chronic inflammatory liver phenotype. Low-grade inflammation could potentially explain the increased VWF content detected in the liver endothelial cells.

Our finding that *F8* deficiency leads to increased plasmatic VWF levels in mice is in line with the published report that haemophilia A patients that were injected with FVIII concentrate showed reduced VWF plasma levels [20]. Also, the involvement of FVIII in the control of VWF plasma levels could be clearly demonstrated as substituting *F8*^*-/y*^ mice with recombinant human FVIII led to lowering of VWF plasma levels. In contrast to the biochemical *in vitro* studies that have demonstrated a synergistic role of FVIII and platelets on the cleavage of VWF multimers by ADAMTS13 [15–17], the increased VWF plasma levels measured in the *F8*-deficient C57BL/6J mouse line were not associated with altered VWF multimer patterns.

Our results clearly demonstrate that *in vivo* inhibition of sialylation with the global sialyltransferase inhibitor 3F-NeuAc reduced VWF antigen levels in the plasma of *F8*^*-/y*^ mice. However, since we did not detect differences in the overall sialylation of plasma proteins comparing *F8*^*+/y*^ WT mice with *F8*^*-/y*^ mice, the unchanged multimer pattern along with increased VWF plasma levels could not be explained by the increased hepatic expression levels of the modifier gene ST3Gal4 in *F8*^*-/y*^ mice [21]. Therefore, the increased VWF content in the liver endothelium and the elevated plasma levels are likely resulting from hepatic low-grade inflammation in *F8*^*-/y*^ mice. Based on the elevated inflammatory markers in *F8*^*-/y*^ mice, it will be interesting to pinpoint the role of FVIII in the regulation of low-grade inflammation of the liver. In contrast to clinical hemophilia A studies, which due to patient heterogeneity and ongoing substitution therapy are prone to confounding factors, the established mouse model could be instrumental to study effects of *F8*-deficiency on inflammatory phenotypes and associated co-morbidities of the disease.

## Supporting information

S1 Fig Blood cell counts of *F8*^*+/y*^ (WT) vs *F8*^*-/y*^ mice. (**A**) White blood cell counts (n = 5). (**B**) red blood cell counts (n = 5). (**C**) hemoglobin levels (n = 5). (**D**) platelet counts (n = 5). (**E**) Neutrophil counts (n = 5). (**F**) Lymphocyte counts (n = 5). (**G**) Monocyte counts (n = 5). (**H**) Eosinophil counts (n = 5). (**I**) Basophil counts (n = 5). All data were expressed as means ± SEM. Statistical comparisons were performed using the Student’s *t*-test.
(TIF)

S2 Fig Hepatic and lung mRNA levels of VWF clearance and sialylation genes in *F8*^*+/y*^ (WT) vs *F8*^*-/y*^ mice. Hepatic transcript levels of (**A**) CLEC4M (n = 5,3), (**B**) ASGR1 (n = 5,4), (**C**) ASGR2 (n = 7), (**D**) SiglecF (n = 3,5), (**E**) ST3Gal4 (n = 5,7). Lung transcript levels of (**F**) LRP1 (n = 6), (**G**) ST3Gal4 (n = 5,6) and (**H**) VWF (n = 7). All data were expressed as means ± SEM. Statistical comparisons were performed using the Student’s *t*-test, * p< 0.05.
(TIF)

S3 Fig Treatment of *F8*^*+/y*^ (WT) and *F8*^*-/y*^ mice with global sialylation inhibitor, 4,7,8,9-pentaacetyl-3Fax-Neu5Ac-CO2Me (3F-NeuAc). (**A**) VWF plasma levels and (**B**) sialic acid plasma levels of untreated *F8*^*+/y*^ (WT) (n = 9,16) and *F8*^*-/y*^ mice (n = 9,10) vs WT intravenously injected with vehicle control (DMSO) (n = 6) and *F8*^*-/y*^ mice i.v. treated with 100mg/kg body weight 3F-NeuAc (Inhibitor) (n = 4), respectively. All data were expressed as means ± SEM. Statistical comparisons were performed using one-way ANOVA, * p< 0.05, ** p<0.01.
(TIF)
